# A Rare Case of Metastatic Gastric Signet Ring Cell Adenocarcinoma in a 23-Year-Old Female Presenting as Malignant Pleural Effusion

**DOI:** 10.7759/cureus.33085

**Published:** 2022-12-29

**Authors:** Beatrice E Torere, Henry O Aiwuyo, Nosakhare Ilerhunmwuwa, Hafiz M Raza, Jiahuai Tan, Tatiana Belousova, Mustafa Wasifuddin

**Affiliations:** 1 Internal Medicine, North Mississippi Medical Center, Tupelo, USA; 2 Internal Medicine, Brookdale University Hospital Medical Center, Brooklyn, USA; 3 Oncology, North Mississippi Medical Center, Tupelo, USA; 4 Pathology, North Mississippi Medical Center, Tupelo, USA

**Keywords:** signet ring cell carcinoma, pleural effusion, metastatic gastric adenocarcinoma, malignant pleural effusion, gastric cancer

## Abstract

Signet ring cell carcinoma (SRCC) is a poorly differentiated mucin-producing adenocarcinoma with greater than 50% signet ring cells. It commonly arises from the gastrointestinal (GI) tract and rarely from extraintestinal organs. This is a rare case of a young African American female who presented with metastatic spread of signet ring cell gastric cancer (pleural and lymph nodal involvement) as the initial presentation of SRCC. Knowledge of the various clinical manifestations of SRCC can help with its early diagnosis, and there is a high need for detailed physical examination, early referral, and prompt treatment in patients with SRCC.

## Introduction

Signet ring cell carcinoma (SRCC) is a poorly differentiated mucin-producing adenocarcinoma with greater than 50% signet ring cells [1]. It commonly arises from the gastrointestinal (GI) tract and rarely from extraintestinal organs [1]. According to the World Health Organization (WHO) global cancer database [2], gastric cancer is the fifth leading cause of cancer and the third cause of cancer death worldwide. The American Cancer Society estimates about 26,380 new cases of gastric cancer in 2022, of whom about 11,090 are expected to die [3]. The Incidence of gastric adenocarcinoma has decreased since the inception of *Helicobacter pylori* (*H. pylori*) eradication [4,5]. However, SRCC incidence increased 10-folds between 1970 and 2000 [6]. A recent study reports that SRCC accounts for 35%-40% of cases of gastric adenocarcinoma and 1% of colorectal cancers [7]. SRCC is an aggressive carcinoma and carries a poor prognosis [8]. The WHO classifies SRCC as a poorly cohesive carcinoma [9], corresponding to the diffuse type of Lauren classification [10] and the undifferentiated type of Nakamura et al. classification [11]. SRCC comprises individual tumor cells invading the surrounding tissues with no gland formation [1].

SRCC is generally seen in adults over 30 years [8,12], unlike familial gastric cancer, which is common in patients less than 30 years old [13]. Multiple reports indicate that younger females are more affected, and more cases are reported among Asian, African, Hispanic, Pacific Islander, and Native Alaskan populations [7,8,12]. The exact cause of SRCC is unknown. SRCC develops under the influence of several genetic and environmental factors [1,8,12]. Genetic mutations such as E-cadherin (CDH 1) have been implicated in developing signet ring cancer [12].

Approximately 15% of all cancer patients develop pleural effusion, with lung and breast cancer accounting for 50%-60%, followed by mesothelioma, lymphoma, and other hematologic malignancies [14,15]. SRCC more frequently metastasizes within the peritoneum, bone, and ovaries and less frequently to the lungs and liver than other adenocarcinomas [1,8,12].

We present a rare case of a young African American female who presented with metastatic spread of signet ring cell gastric cancer (pleural and lymph nodal involvement) as the initial presentation.

## Case presentation

Our patient is a 23-year-old African American female with a past medical history of tobacco abuse who presented to the emergency department (ED) with complaints of cough, chest pain, diarrhea, and abdominal pain of three days duration. She has a family history of breast cancer in her maternal grandmother. Upon arrival at the ED, she was tachycardic and febrile with a temperature of 100.4 degrees Fahrenheit. Her physical examination was significant for left cervical and right inguinal lymphadenopathy with decreased breath sounds in bilateral lung bases. Laboratory investigation was remarkable for positive influenza B virus, anemia, normal white blood cell count, negative stool culture, and no pulmonary infiltrates on chest X-ray (Table 1).

**Table 1:** Findings of the complete blood count WBC = white blood cell; MCV = mean corpuscular volume; MCH = mean corpuscular hemoglobin; MCHC = mean corpuscular hemoglobin concentration; RDW = red blood cell distribution width; MPV = mean platelet volume; ↔ = within normal limit; ↓ = below normal limit; ↑ = above normal limit

Test	Finding	Reference range
WBC count (mL/μL)	4.46 ↔	4-5.5
Hemoglobin (g/dL)	11.2 ↓	12-16
Hematocrit (%)	37.7 ↔	37-47
MCV (FL)	84.5 ↔	82-96
MCH (PG)	25.1 ↓	27-32
MCHC (g/dL)	29.7 ↓	32-36
RDW (%)	14.4 ↔	12-15.4
Platelet count (×1,000/μL)	245 ↔	150-400
MPV (FL)	11.8 ↔	9.2-12.8
Neutrophils (%)	54 ↔	50-70
Lymphocyte (%)	27 ↔	20-40
Monocyte (%)	13 ↑	2-8
Eosinophil (%)	6 ↑	0-3
Basophil (%)	0 ↔	0-1

**Figure 1:**
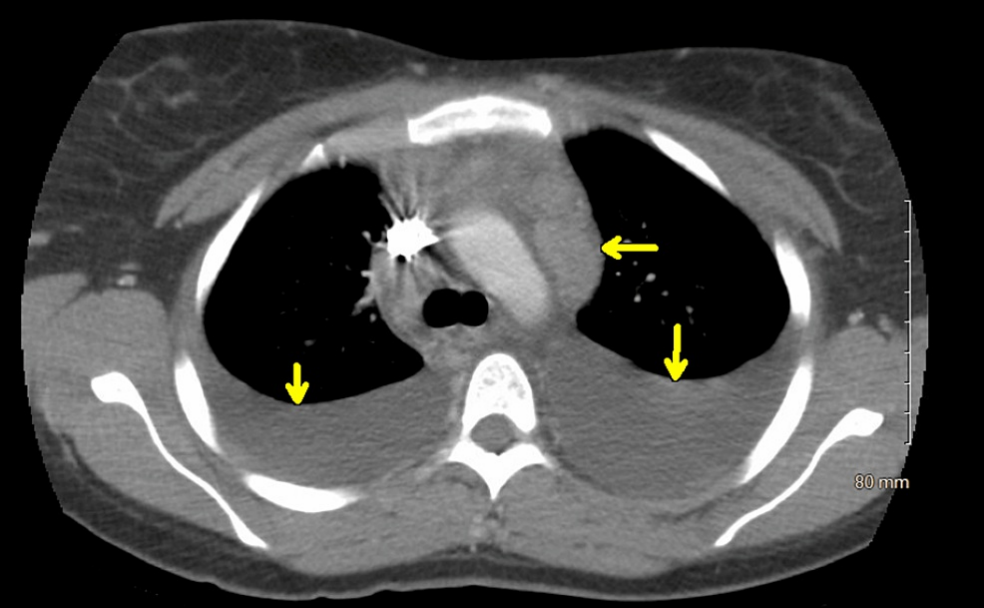
Computed tomography scan image of the chest with bulky intrathoracic adenopathy Arrows indicate bulky intrathoracic adenopathy.

The patient was admitted, and computed tomography (CT) of the chest done in the hospital showed bulky intrathoracic adenopathy (Figure 1), index node within the right infra-hilar region, extending into the subcarinal nodal station, measuring 1.8 centimeters (cm) in short axis, along with moderate to large bilateral pleural effusions. CT of the abdomen and pelvis revealed a small to moderate amount of free fluid throughout the abdomen and pelvis and retroperitoneum with a suggestion of inflammatory bowel wall thickening involving the right colon.

**Figure 2:**
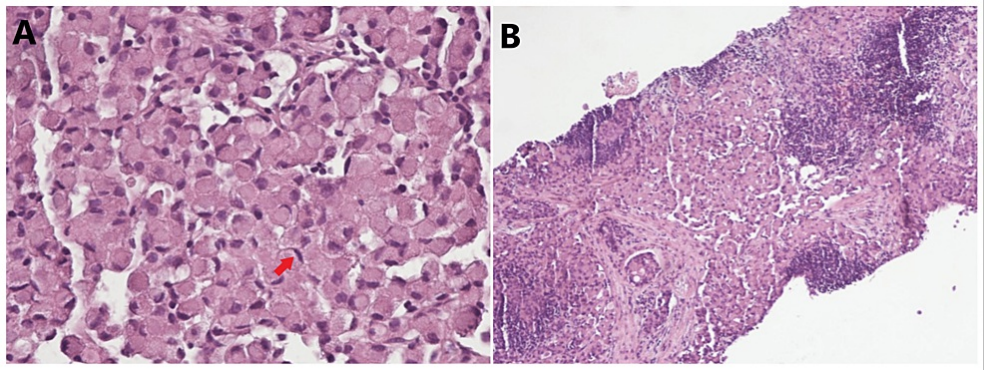
Microscopic image of the lymph node biopsy showing lymphoid tissue with malignant infiltrate by poorly cohesive pleomorphic signet ring cell carcinoma with prominent lymphovascular invasion within desmoplastic reaction (hematoxylin and eosin stain) A = ×400; B = ×100 Arrow indicates signet ring cells in the lymph node.

**Figure 3:**
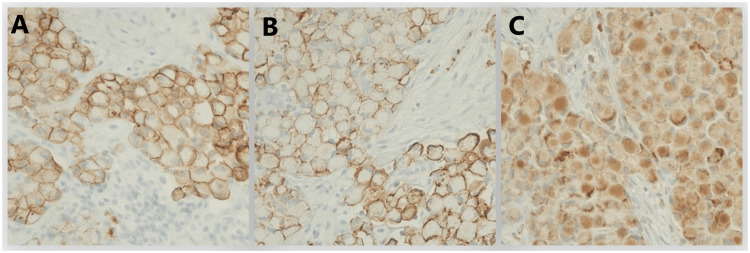
Immunohistochemistry results showing positive tumor marker activity A = E-cadherin; B = CD138; C = CD68 CD = cluster of differentiation

**Figure 4:**
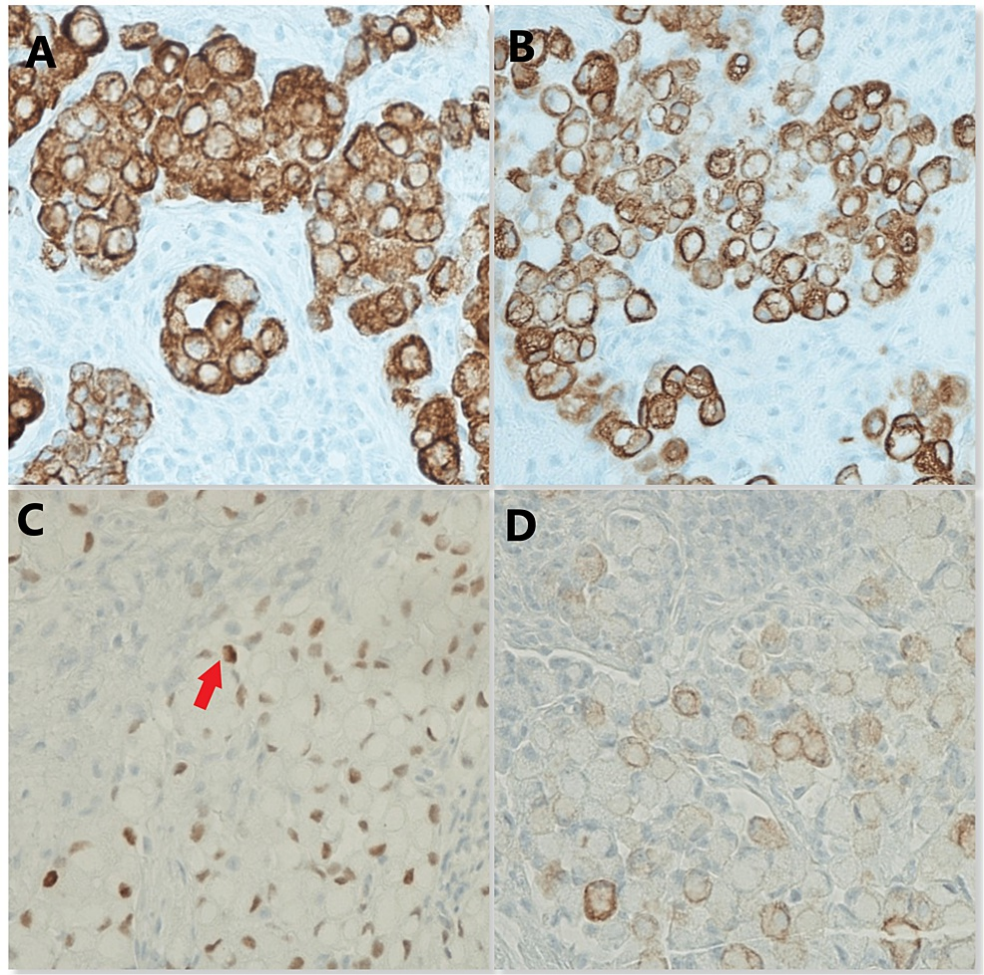
Immunohistochemistry stain results showing positive gastrointestinal tract-specific tumor markers A = CK8/18; B = CK20; C = CDX2; D = CK7 CK = cytokeratins; CD = cluster of differentiation Arrow indicates the signet ring cell.

The patient underwent bronchoscopy with endobronchial ultrasound and thoracentesis. Pleural fluid cytology was benign. A left supraclavicular lymph node biopsy was obtained. She received supportive treatment with intravenous Zofran (ondansetron) and analgesics. The pathology of the left supraclavicular node biopsy (the patient declined biopsy of the inguinal node) showed lymphoid tissue with malignant infiltration by poorly cohesive signet ring cells with prominent lymphovascular invasion (Figures 2-4).

**Figure 5:**
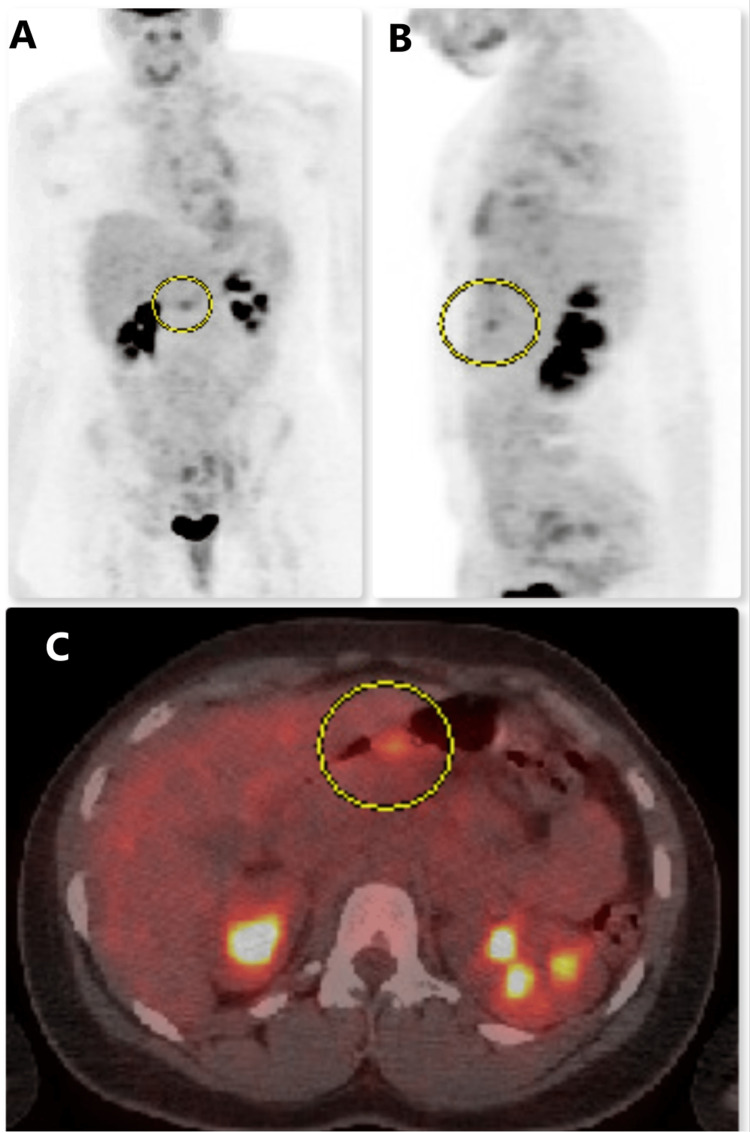
PET scan showing cancer cells in the stomach A = frontal view; B = longitudinal view; C = transverse view PET = positron emission tomography Circle markers indicate cancer cells.

The patient was also referred to oncology, where a positron emission tomography (PET) scan showed a primary gastric tumor (Figure 5).

**Figure 6:**
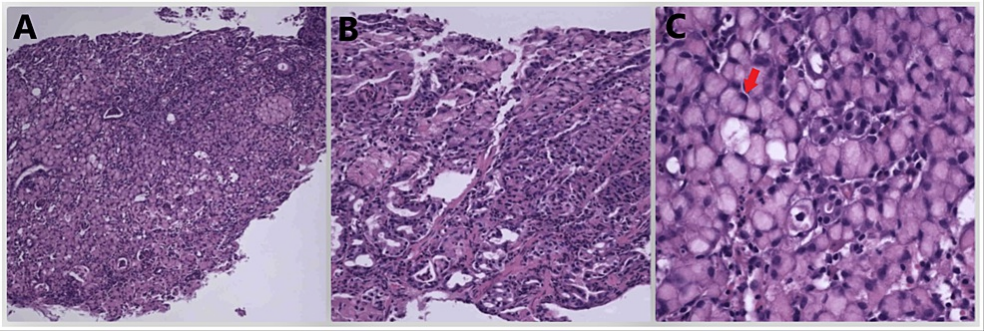
Microscopic image of the poorly differentiated adenocarcinoma (signet ring cell type) in the stomach (hematoxylin and eosin stain) A = ×10; B = ×20; C = ×40 Arrow indicates the signet ring cell.

Esophagogastroduodenoscopy revealed erythematous mucosa in the stomach, which was biopsied and was pathology consistent with poorly differentiated adenocarcinoma (signet ring cell type) identified within gastric antral-type mucosa with *Helicobacter pylori*-associated moderately active chronic gastritis identified within gastric antral and fundic (oxyntic) mucosa (Figure 6). No intestinal metaplasia was identified.

Cancer antigen 125 was elevated at 71.2 units per milliliter, and cancer antigens 19-9 and 15-3 were within normal limits. Genetic testing revealed negative human epidermal growth factor receptor 2 (HER 2), negative programmed cell death ligand 1 (PDL1) expression, and negative targeted mutation on genomic testing (Tempus tumor test). She was diagnosed with stage 4 signet cell gastric adenocarcinoma. Oncology recommended chemotherapy and immunotherapy with 5-fluorouracil, oxaliplatin, and nivolumab; however, the patient declined recommendations. She was hospitalized for symptomatic pleural effusion two weeks after the clinic visit. Pleural fluid cytology was suspicious for metastatic adenocarcinoma; she refused chemotherapy. She was readmitted for recurrent pleural effusion; a pericardial window was placed, plus right video-assisted thoracoscopic surgery with talc pleurodesis. At this point, she accepted chemotherapy and received the first dose inpatient. The patient received five cycles of the combination therapy of leucovorin calcium (folinic acid), fluorouracil, and oxaliplatin (commonly called FOLFOX) and nivolumab. However, she continued to decline in her functional status with multiple hospitalizations for recurrent malignant pleural effusion. Palliative care was consulted. She opted for a Do Not Resuscitate (DNR) code status and home hospice care.

## Discussion

Signet ring cell carcinoma (SRCC) is an aggressive and poorly differentiated gastric adenocarcinoma that occurs in the stomach in 90% of cases [1,8,12]. Patients with early SRCC may not have clinical symptoms and are typically in the advanced stage when they present [12]. Many cases of gastric cancers are likely to present initially with nonspecific gastrointestinal (GI) symptoms and may be misdiagnosed [1,8,12]. SRCC commonly presents with indigestion, dysphagia, nausea, vomiting, abdominal pain, postprandial fullness, anorexia, GI bleeding, pallor, fatigue, and joint pain as the initial presentation because of its common areas of metastases including the peritoneum, bone, and ovaries [7,8,12]; it rarely involves the lung or pleural. Our patient presented with malignant pleural effusion, bulky mediastinal involvement of the lymph nodes, and evidence of ovarian metastasis (elevated cancer antigen 125). She had Virchow’s nodes, which is the presence of left-sided supraclavicular lymphadenopathy, as the initial presentation of underlying gastric adenocarcinoma. The prognosis of malignant pleural effusion presentation is poor [14,15].

SRCC is more associated with genetic mutations in the CDH 1 gene, which is necessary for maintaining E-cadherin integrity [12]. Mutations in this gene lead to loss of cohesiveness of the epithelial lining of the transformed cells, increasing the chances of early metastasis. Although our patient had a family history of prostate cancer in her paternal grandfather and breast cancer in her maternal grandmother, Tempus did not report any targetable mutations. Also unique to our case is the rare occurrence of moderate activity of *H. pylori*-associated gastritis in the antral and fundic mucosa. The stomach fundus is not a common site for *H. pylori*-associated inflammation, and the patient had diffuse chronic gastritis, which affects the upper and lower stomach [16,17]. Antral involvement of the SRCC was identified on histology, which may raise a likely possibility of an *H. pylori*-associated SRCC, as reported in recent literature [18]. However, the role of *H. pylori* in SRCC is controversial. Also, our patient had tobacco dependence, which can potentiate the carcinogenic effect of *H. pylori*, especially in patients with a genetic tendency to gastric cancer [19]. However, the role of smoking, obesity, salt-preserved food, or autoimmune gastritis is not well studied in SRCC.

The assessment of molecular characteristics of gastric cancer helps select patients who might benefit from a certain therapy. Currently, there are only three therapeutic-relevant, routinely tested biomarkers in gastric cancer (HER 2 expression, PDL1 expression, and deficient mismatch repair) [20]. Our patient had a negative expression of HER 2, negative PDL1, and stable microsatellites, so she could not benefit from therapies targeting these biomarkers. She was treated with five cycles of FOLFOX and nivolumab, but her cancer was incurable.

This case emphasized the importance of thorough physical examination, including lymph node examination, in all patients and early referral. Our patient’s initial presentation and evaluation results include chest pain, abdominal pain, diarrhea, fever, negative stool studies, bilateral pleural effusion, and benign pleural fluid analysis. However, the sudden onset of large pleural effusion in the setting of left cervical and inguinal lymphadenopathy in a 23-year-old female prompted a lymph node biopsy.

## Conclusions

The rarity of gastric cancer in young adults and its association with nonspecific GI symptoms increase the risk of misdiagnoses in them. Knowledge of the typical and atypical clinical manifestations of SRCC could help physicians with early diagnoses. We emphasize the need for thorough physical examination, early referral, and prompt treatment in patients with SRCC.
